# Cardiovascular Morbidity in Systemic Lupus Erythematosus: A Single-Center Retrospective Study

**DOI:** 10.7759/cureus.57842

**Published:** 2024-04-08

**Authors:** Yousef Alammari, Fahed A Albednah, Khalid A Alharbi, Abdulrahman M Alrashoudi, Abdulaziz Y Alsharif, Abdullah H Alkahtani, Hasan Z Alshehry, Abdulrahman A Alajaji, Ahmed M Alsaedi, Khalid Al harbi, Rayan Abubakker Qutob, Mohammed Almansour

**Affiliations:** 1 Rheumatology, College of Medicine, Imam Mohammad Ibn Saud Islamic University (IMSIU), Riyadh, SAU; 2 College of Medicine, Imam Mohammad Ibn Saud Islamic University (IMSIU), Riyadh, SAU; 3 Medicine and Surgery, College of Medicine, Imam Mohammad Ibn Saud Islamic University (IMSIU), Riyadh, SAU; 4 Internal Medicine, King Fahad Medical City, Riyadh, SAU; 5 Cardiology, College of Medicine, Imam Mohammad Ibn Saud Islamic University (IMSIU), Riyadh, SAU; 6 Internal Medicine, College of Medicine, Imam Mohammad Ibn Saud Islamic University (IMSIU), Riyadh, SAU; 7 Rheumatology, King Fahad Medical City, Riyadh, SAU

**Keywords:** lupus, sle, hypertension, ischemic heart disease, saudi arabia, cardiovascular

## Abstract

Background: Systemic Lupus Erythematosus (SLE) is an autoimmune inflammatory condition affecting multiple systems. Cardiovascular morbidity is a significant concern, with around 25% of SLE patients experiencing cardiac complications.

Objective: This study aims to determine the prevalence of cardiovascular morbidity in SLE patients in King Fahad Medical City (KFMC) in Riyadh, Saudi Arabia.

Methodology: This retrospective record-based research was conducted at KFMC from January 2015 to October 2023. A review of the medical files of all SLE patients was accomplished.

Results: The vast majority of the patients (90.9%) were females. The mean age for the patients was 36.5 years. The most common comorbidities were lupus nephritis (34.6%), hypothyroidism (18.4%), and anti-phospholipid syndrome (9.2%). The most commonly used medications were hydroxychloroquine (81.8%), corticosteroids (prednisolone) (43.0%), and mycophenolate mofetil (27.9%). Around 45.2% (n= 176) of the patients with SLE developed cardiovascular complications. The most commonly reported cardiovascular diseases that developed after diagnosing patients with SLE were hypertension (22.4%), valvular heart diseases (13.2%), and dyslipidemia (9.2%). The study also found that anti-dsDNA antibodies can reduce the likelihood of developing hypertension by 40%. This research contributes to the medical literature on SLE and sets the stage for future research on personalized healthcare strategies for managing SLE and its complications.

Conclusion: This study highlights that a considerable proportion of SLE patients(~50%) develop cardiovascular complications, with hypertension, valvular heart diseases, and dyslipidemia being the most common. We also discovered that anti-double-stranded deoxyribonucleic acid antibodies (Anti-dsDNA) reduce the likelihood of developing hypertension.

## Introduction

Systemic lupus erythematosus (SLE) represents a challenge to all rheumatologists worldwide due to its broad clinical signs. SLE is an autoimmune inflammatory condition frequently affecting multiple systems, including the musculoskeletal, cardiovascular, renal, and brain [1]. Data concerning the epidemiology of Lupus is insufficient; however, some studies have aimed to identify the prevalence of Lupus in different countries and regions. Hence, a study was conducted to determine the global prevalence of SLE. SLE is found worldwide, with varied prevalence rates based on gender, ethnicity, socioeconomic status, age, genetics, and environmental factors. These characteristics influence the disease’s prevalence and outcome, as some are associated with worse results. It is estimated that Saudi Arabia has a ratio of 19.3 per 100,000 people [2].

Due to the early diagnosis of SLE, the overall survival improved from 60% in 1950 to 95% in 2000. Females are more affected than males in early and late-onset SLE, with a ratio of 8-15:1 in early-onset SLE and 2-8:1 in late-onset SLE. Even though females are more affected by SLE, males have a worse prognosis and are at a higher risk of mortality and early organ damage because the clinical phenotypes of SLE in females differ from those in males. SLE patients have a distinct genetic lineage, which impacts the disease’s prognosis. The incidence and prevalence of SLE vary dramatically around the world. The observed variances can be due to genetic, environmental, socio-demographic, and methodological factors yet to be considered regionally [2]. The pathophysiology of SLE is complicated, and our understanding of it is constantly changing. However, it has been suggested that the activation of autoimmunity plays a significant role in SLE development [1,3]. The latest 2019 European League Against Rheumatism (EULAR) and the American College of Rheumatology (ACR) classification criteria are used for the diagnosis of SLE, and the criteria make no distinction between clinical and immunologic criteria; it comprises 10 main items [4,5].

In up to 50% of SLE patients, serious complications like infections develop mainly in the skin, respiratory, and renal systems. Correspondingly, cardiac complications affect the pericardium, myocardium, valves, conduction system, and coronary arteries in people with SLE. It is estimated that the overall percentage of cardiac involvement in SLE is around 25% [5]. The most common presentation of cardiac involvement is pericarditis, which can range from mild effusion to more complicated forms such as cardiac tamponade. The literature has widely ranged pericarditis incidence among SLE patients between 11% to 54%, and a retrospective study done on 180 patients diagnosed with SLE to determine the frequency of pericarditis found that 39% of patients had frequent pericarditis related to pleural effusions. Also, SLE patients occasionally suffer from autoimmune myocarditis, raising the risk of arrhythmia. The studies suggest that immune complex formation is responsible for the pericardium’s and myocardium’s inflammatory processes, and anti-Smith (anti-Sm) antibodies correlate with pericarditis [5-8].

A less common cause of cardiac involvement is sterile endocarditis (Libman-Sacks syndrome), which most commonly affects the mitral valve. Raynaud’s syndrome, vasculitis, and arterial and venous thrombosis can all develop, especially when antiphospholipid syndrome is present. Above all, coronary artery disease (CAD) and ischemic heart disease (IHD) are the most common cause of mortality in elderly patients with SLE [5,9]. Besides, those diagnosed with SLE have between 9 and 50-fold more chances of developing myocardial infarction than the normal population [10].

Likewise, patients with SLE have a higher risk than normal individuals of developing hypertension; the numbers showed that 74% of patients with Lupus suffer from hypertension. The results have discovered that SLE patients, regardless of their demographic, with a constant BP reading of > 130/80, when observed for a two-year interval, delineated a higher cardiovascular event incidence (HR 1.73, 95% CI 1.13 to 2.69 p=0.011). As a result, the target blood pressure of all patients diagnosed with SLE should be less than 130/80 [11].

Additionally, a study investigated the incidence and risk of heart failure (HF) in SLE patients. The finding of this study suggests that those suffering from SLE have a significantly higher risk of developing HF and a worse cardiovascular risk profile compared with the general population [12]. Furthermore, it was found that patients with a history of SLE and heart failure have a higher mortality risk than those with heart failure only (adjusted hazard ratio: 1.50; 95% CI: 1.08 to 2.08) [13].

Moreover, atherosclerosis is considered an important major cause of mortality in patients with SLE; studies have shown that the prevalence of atherosclerosis in patients with SLE is higher compared to the normal population. A study compared SLE patients to matched controls, and the results showed an increase in the prevalence of atherosclerosis among SLE patients than in the control group (37.1% vs. 15.2%, P<0.001) [14]. Notably, a study discovered that more than 80% of myocardial infarction among SLE patients was associated with coronary atherosclerosis and preceded by symptomatic CAD [14]. In addition, SLE-specific risk factors, such as glucocorticoid therapy, antiphospholipid antibodies, and renal disease, may have a role in developing atherosclerosis in SLE patients [14-17]. Another study found that the presence of anti-double-stranded deoxyribonucleic acid antibodies (Anti-dsDNA) raises the chance of cardiovascular complications among SLE patients [18]. Kidney involvement can be seen clinically in about 50% of SLE patients, confounding comorbidity that may contribute to cardiovascular morbidity and mortality [19-20].

For this reason, this study aims to determine the prevalence of cardiovascular morbidity in SLE patients in KFMC in Riyadh, Saudi Arabia, to find an association between cardiovascular morbidity and SLE, and to determine any association between SLE-specific antibodies and cardiovascular complications. These associations may differ from worldwide estimates due to reasons including socioeconomic factors, lifestyle factors, and public health, which will be considered in this study.

## Materials and methods

Study design

This is a retrospective record-based research study conducted at KFMC by reviewing medical files from January 2015 to October 2023, as we could only access data from 2015 recorded in the Epic system of KFMC. A thorough review of the medical files of all SLE patients was accomplished and a convenient sampling technique was implemented. The minimum sample size needed to achieve a precision of ±5% with a 95% confidence interval was 385 subjects.

Data collection tool

The inclusion criteria for participants in our study was diagnosis with SLE by a physician. The exclusion criteria were patients younger than 16 years old, patients who are to be considered a vulnerable group per Institutional Review Board (IRB) recommendations, patients whose data are incomplete/invalid, and patients who wish to withdraw from the research as per their wish.

Our research collected the data by accessing the patient’s medical records after gaining permission. We focused on the cardiovascular risks and morbidities in SLE patients, such as hypertension, dyslipidemia, valvular heart diseases, myocardial infarction, and pericardial diseases, depending on the routine screening of these diseases by laboratory findings, including cardiac enzymes such as Troponins T, creatine kinase (CK) and lactate dehydrogenase (LDH), echocardiography (both esophageal and transthoracic), and electrocardiography. All participants received the same complete cardiovascular examination to avoid the possibility of cardiovascular disease being missed in some subjects due to an incomplete examination and affecting the results of the study. In addition, we have collected data about medications that the patient has been using, such as azathioprine, hydroxychloroquine, cyclophosphamide, corticosteroids, methotrexate, aspirin, monoclonal antibodies, Mycophenolate mofetil, statins, and non-steroidal anti-inflammatory drugs (NSAIDs). Furthermore, we studied the relationship between cardiovascular diseases in SLE patients and their lupus antibodies profile. We also collected data that could be related to cardiovascular risk and morbidity in SLE patients, such as patients with diabetes mellitus, hypothyroidism, lupus nephritis (LN), and smoking.

Ethical consideration

Based on the declaration of Helsinki, IRB approval was obtained from the IRB unit, KFMC, Riyadh, Saudi Arabia (Approval number: FWA00018774) on October 1, 2023. The IRB approval waived patient consent since this is a retrospective study and data was collected through the patient's medical records.

Statistical analysis

Statistical package for social science software, version 29 (IBM SPSS Statistics 29.0), was used to analyze the data for this study. Descriptive statistics was used to present categorical variables. Categorical variables were presented using frequencies and percentages. The normality of continuous variables was checked using histogram, skewness, and kurtosis measures. Continuous variables were presented using the mean and standard deviation. The imputation technique was used to complete the missing data. Binary logistic regression analysis was used to identify the association between the presence of antibodies (independent variables) and the development of cardiovascular diseases after diagnosing SLE (dependent variable). The odds ratios were presented with a 95% confidence interval. The significance level was assigned as a p-value below 0.05.

## Results

A total of 390 patients were involved in this study. The vast majority of the patients (90.9%) were females. The mean age for the patients was 36.5 years (standard deviation: 12.7) and ranged between 16 and 72 years. The mean disease duration was 8.1 years (standard deviation: 3.8) and ranged between 1 and 27 years. The most common comorbidities were lupus nephritis (34.6%), hypothyroidism (18.4%), and anti-phospholipid syndrome (9.2%). Table 1 presents the baseline characteristics of the patients.

**Table 1:** Patients’ baseline characteristics.

Variable	Frequency	Percentage
Gender		
Females	361	90.9%
Mean age (standard deviation) years	36.5 (12.7)	
Mean duration of disease (standard deviation) years	8.1 (3.8)	
Comorbidities		
Lupus nephritis	135	34.6%
Hypothyroidism	73	18.4%
Anti-phospholipid syndrome	36	9.2%
Diabetes mellitus	31	7.9%
Chronic kidney disease	24	6.2%
Epilepsy	23	5.9%
Hypertension	12	3.1%
Asthma	11	2.8%
Rheumatoid arthritis	10	2.6%
Avascular necrosis	4	1.0%

**Figure 1:**
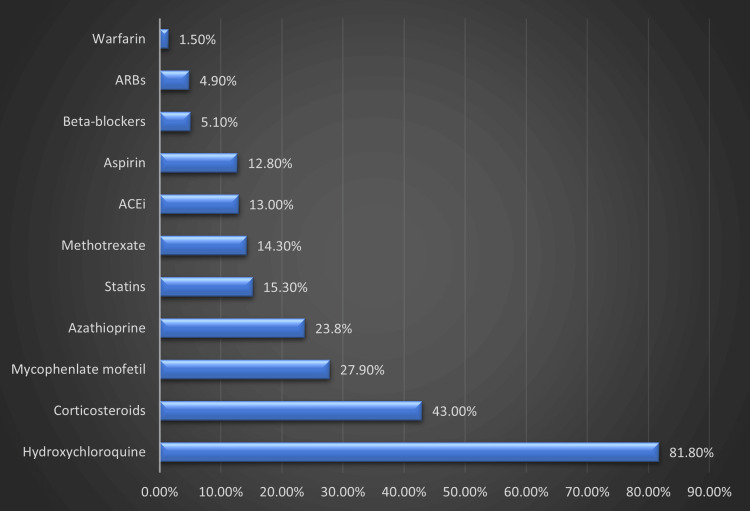
Medications use profile

Figure 1 displays the medication use history among the patients examined in this study. The most commonly used medications were hydroxychloroquine (81.8%), corticosteroids (prednisolone) (43.0%), and mycophenolate mofetil (27.9%).

**Figure 2:**
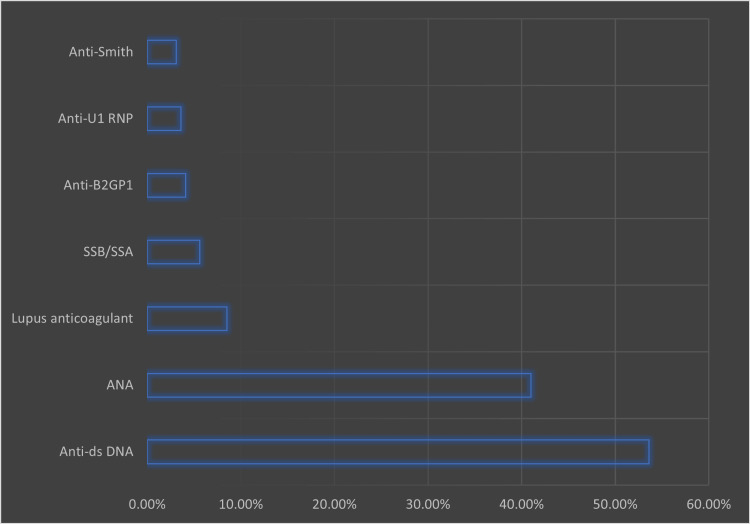
SLE Patients’ antibody profile

Figure 2 presents the patients’ antibody profiles. The most prevalent antibodies among patients with SLE were anti-ds DNA (53.6%), antinuclear antibody (ANA) (41.0%), and lupus anticoagulant antibodies (8.5%).

**Figure 3:**
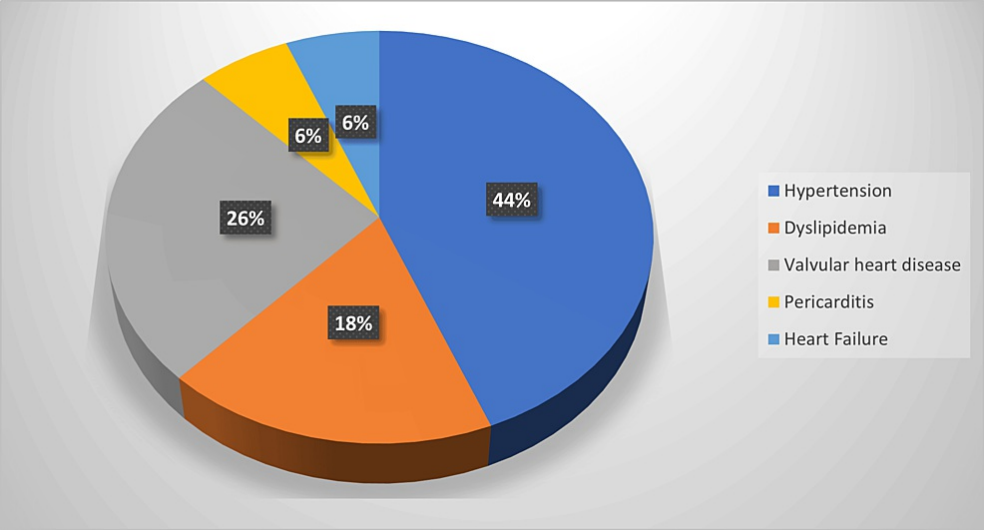
Cardiovascular complications among SLE patients

Figure 3 shows the cardiovascular diseases among the study participants after SLE diagnosis. Around 45.2% (n= 176) of the patients with SLE developed cardiovascular complications. The most commonly reported cardiovascular diseases that developed after diagnosing the patients with SLE were hypertension (22.4%), valvular heart diseases (13.2%), and dyslipidemia (9.2%). Concerning patients with lupus nephritis, around (31.1%) of the patients developed hypertension, (21.1%) developed valvular heart diseases, (11.9%) developed dyslipidemia, (5.9%) developed heart failure, and (3.0%) developed pericarditis.

Binary logistic regression analysis identified that the presence of Anti-ds DNA decreases the likelihood of developing hypertension by 40% (odds ratio: 0.60 (95% confidence interval (0.37-0.97)), (p<0.05) (Table 2).

**Table 2:** Binary logistic regression analysis

SLE antibodies	Odds ratio of developing cardiovascular disease				
	Hypertension	Dyslipidemia	Valvular heart disease	Pericarditis	Heart failure
SSB/SSA	0.16 (0.02-1.17)	0.45 (0.06-3.47)	1.50 (0.49-4.62)	1.54 (0.19-12.51)	-
Anti-U1 RNP	0.95 (0.26-3.47)	-	-	-	-
Anti-B2GP1	1.61 (0.55-4.78)	0.65 (0.08-5.04)	0.43 (0.06-3.30)	-	-
Lupus anticoagulant	1.12 (0.49-2.59)	0.61 (0.14-2.67)	1.19 (0.44-3.24)	0.98 (0.12-7.84)	0.98 (0.12-7.86)
Anti-ds DNA	0.60 (0.37-0.97)*	1.09 (0.55-2.18)	1.42 (0.78-2.59)	0.61 (0.19-1.96)	0.61 (0.19-1.95)
ANA	0.74 (0.45-1.22)	0.80 (0.39-1.62)	1.20 (0.66-2.18)	0.71 (0.21-2.39)	0.71 (0.21-2.40)
Anti-Smith	-	-	-	-	-

## Discussion

The association between SLE and cardiovascular complications has been well-established over the years. However, the variety of these complications has been a gray zone in medical literature, especially in Saudi Arabia [1-5]. Our study explored the variation of these cardiovascular complications and whether there are associations with other confounding factors that may precipitate these complications.

In our study, the prevalence of cardiovascular complications among patients with SLE was found to be 45.2%. The specific disorder included hypertension (44%), valvular heart disease (26%), dyslipidemia (18%), pericarditis (6%), and heart failure (6%). In comparison, a similar local study reported a prevalence of cardiovascular complications at 18%, with specific diseases including pericarditis and myocarditis (14%), endocarditis (11%), myocardial infarction (20%), coronary artery disease (38%), and valvular heart diseases (14%) [21].

Initially, the most observed medical disorders in our population, as aforementioned, were lupus nephritis (LN), hypothyroidism, and antiphospholipid syndrome, all of which have been previously associated with cardiovascular morbidity either directly or indirectly [22-24].

Furthermore, our study reports additional comorbidities that are recognized for elevating the risk of cardiovascular complications, including diabetes (7%) and chronic kidney disease (6%). Cardiovascular disorders varied amongst SLE patients; notable entities that were observed include hypertension, valvular heart disease, dyslipidemia, heart failure, and pericarditis (Figure 3). The prevalence of valvular heart disease in SLE patients is high, with mitral regurgitation accounting for most cases, followed by tricuspid regurgitation [25]. Our study found that 7% of our patients were diagnosed with mitral regurgitation and 5% with tricuspid regurgitation. Compared to different studies, the prevalence was more than 19% and 10%, respectively [26]. Cardiovascular complications in SLE often present with no symptoms or murmur, which could hinder the diagnosis [27]. With such a high association rate and vague presentation, many studies have suggested screening using echocardiograms for early detection [26].

A systematic review and meta-analysis were performed to assess the cardiac abnormalities based on the changes in the echocardiography in patients with SLE. The study found that patients with SLE developed cardiac abnormalities, including pericardial effusion, with the highest rate and risk when compared with healthy controls, followed by combined valvular alterations and left atrial and ventricular abnormalities with an increase in left atrial diameter (LAD), left ventricular internal diameter in diastole (LVDd), and left ventricular mass index (LVMI), and a decrease in the left ventricular systolic function and diastolic function. This study suggests that echocardiographic assessment should be considered part of a routine examination for SLE patients, especially those with a long history of SLE (>10 years) or active disease parameters, and even newly diagnosed ones [27]. Additionally, many studies suggest that there is an increased risk of IHD in patients with SLE (10-fold risk compared to the normal population). The prevalence of IHD in SLE patients is between 3.8 and 16%. In our results, IHDs were 0.8% [28].

Furthermore, LN has been shown to cause a variety of cardiovascular complications directly [22]; this has been reinforced in our research, where LN patients consequently developed hypertension, valvular heart disease, heart failure, pericarditis, and dyslipidemia.

Autoantibodies seen in SLE have been commonly related to diagnosing the disease and, to some extent, the disease severity [29]. We noted that 53% of the total study population had anti-dsDNA, followed by ANA and lupus anticoagulant (Figure 2). Regression analysis indicates that anti-dsDNA-positive SLE patients are less likely to develop hypertension with a risk reduction of 40% (0.6 OR; 95% CI 0.37-0.97; p <0.05). However, another study found that anti-dsDNA in SLE increases cardiovascular morbidity risk [18].

Therapeutic interventions for the SLE patients who participated in this study consisted of only medical therapy, namely with hydroxychloroquine, the primary long-term therapy affirmed by the consensus in the medical field [30]; approximately 81% of patients have been or are still undergoing therapy. Other medications include prednisolone and mycophenolate mofetil, both of which have been prescribed mainly as targeted therapy for LN [31].

Limitations

It is crucial to recognize and address the inherent limitations that may impact the generalizability and scope of our findings. Firstly, retrospective studies limit researchers from accessing real-time data, which could affect its accuracy. Secondly, the nature of collecting data from medical records and how it is obtained exposes the data to recall bias, as documentation can vary. Our sample size was drawn from a single medical center in Riyadh, potentially impacting its accuracy. A more comprehensive sample from the entire region of Saudi Arabia would provide a more accurate assessment.

## Conclusions

This study highlights that 45.2% of SLE patients develop cardiovascular complications, with hypertension, valvular heart diseases, and dyslipidemia being the most common. We also discovered that anti-dsDNA decreases the likelihood of developing hypertension by 40%. In essence, our article enriches the medical literature on SLE, particularly concerning cardiovascular morbidity, and it sets the stage for future research focused on personalized and region-specific healthcare strategies for managing SLE and its complications. The findings serve as a critical resource for rheumatologists, cardiologists, and healthcare professionals involved in the care of SLE patients, emphasizing the need for heightened vigilance and proactive management of cardiovascular risks in this population, especially in the context of Saudi Arabia, where such data has been notably sparse, and underscores the need for region-specific clinical investigations to optimize patient outcomes.
